# Red neurons in ovine polioencephalomalacia (cerebrocortical necrosis) are strongly amyloid precursor protein immunopositive

**DOI:** 10.1007/s11259-022-09888-6

**Published:** 2022-01-21

**Authors:** John W. Finnie, Ian V. Jerrett, Jim Manavis

**Affiliations:** 1 Discipline of Anatomy and Pathology, Adelaide Medical School, University of Adelaide, Adelaide, SA Australia; 2 Agriculture Victoria Research, AgriBio Centre, Bundoora, VIC Australia

**Keywords:** Polioencephalomalacia, Sheep, Red neurons, Amyloid precursor protein immunolabelling, Neuroprotection

## Abstract

The signature pathological feature of the pseudolaminar cerebrocortical necrosis found in polioencephalomalacia (PEM) of ruminants is the development of red (eosinophilic) neurons. These neurons are generally considered to be irredeemably injured but we have shown, for the first time, in ovine PEM cases, that most strongly express amyloid precursor protein (APP), which has a neuroprotective role in the brain. By contrast, neurons in unaffected cerebral cortices from control sheep were APP immunonegative. This finding suggests that, rather than being inevitably destined to die, some of these APP immunoreactive cortical neurons may survive and regain structural and functional integrity.

Ruminant PEM (sometimes termed cerebrocortical necrosis) is an important neurological disorder of sheep, goats, and cattle, being clinicopathologically similar in all of these species, but of generally shorter clinical duration in the first two (Summers et al. 1995; Vandevelde et al. 2012; Cantile and Youssef 2016). It is usually attributed to thiamine deficiency, and can be reproduced by the administration of thiamine antagonists such as amprolium. Thiamine destruction can be induced by thiaminases produced by rumen microbes and present in ingested bracken (*Pteridium* sp.) and nardoo (*Marsilea* sp.) ferns, or the consumption of excess sulphur compounds (Summers et al. 1995; Vandevelde et al. 2012; Cantile and Youssef 2016).

In PEM, there is softening (malacia) of the neocortical grey matter, in particular the territory supplied by the middle and caudal cerebral arteries. The cortical necrosis is pseudolaminar in distribution, involving more than one cortical layer, and damaged neurons show an increased affinity for the acid dye, eosin, in routine haematoxylin and eosin (H&E)-stained brain sections (Summers et al. 1995; Vandevelde et al. 2012; Cantile and Youssef 2016). This tinctorial appearance has led to these neurons being designated “eosinophilic (acidophilic) neurons”, “pink neurons”, and “red neurons” (Kalaria et al. 2015; Vinters and Kleinschmidt-DeMasters 2015) and, while the characteristic morphological feature of PEM, is not pathognomonic for this disease. The term “ischaemic neurons” is now discouraged, especially as this microscopic appearance can be produced by factors other than diminished vascular perfusion (Kalaria et al. 2015; Vinters and Kleinschmidt-DeMasters 2015). Red neurons have a shrunken, hypereosinophilic cytoplasm with nuclear hyperchromasia or pyknosis and the perinuclear clear space is enlarged due to abutting swollen astrocytic processes.

Neuronal red cell change is often considered to be an irreversible morphological alteration (Vandevelde et al. 2012) and these neurons are sometimes referred to as “red-dead” neurons (Vinters and Kleinschmidt-DeMasters 2015). However, the reversibility or otherwise of red neurons is still actively debated (Zille et al. 2012) and there are currently no reliable markers to determine the first irreversible, “point-of-no-return” step in the molecular cascade at which such an injured cell will eventually die (Zille et al. 2012).

While routine H&E staining can provide some useful information regarding the nature of red neurons, such histological interpretations are limited. Accordingly, we used amyloid precursor protein (APP) immunohistochemistry to further investigate neuronal red cell change in ovine PEM, particularly as APP is known to be neuroprotective (Hefter and Draguhn 2017), and its therapeutic administration could potentially mitigate this degenerative change (Plummer et al. 2016; Mockett et al. 2017).

APP is a constitutively expressed, highly conserved, type-1 transmembrane glycoprotein, which is most abundantly expressed in neurons and glia of the central nervous system. It is found in the somatodendritic and axonal compartments of neurons, and in the presynaptic zone, the latter being reached by fast axoplasmic transport. APP is an acute phase protein, promoting survival under metabolically challenging conditions that produce cellular stress, such as ischaemia-hypoxia, traumatic brain injury, and inflammation (Mattson 1997; Finnie et al. 2010; Kogel et al. 2012; Plummer et al. 2016; Hefter and Draguhn 2017). Upregulation of APP in neurotrauma has been found to be due to increased mRNA expression (Van den Heuvel et al. 1999).

APP cleavage by secretases can produce both beneficial and harmful effects. Under physiological conditions, the non-amyloidogenic pathway is dominant, with α-secretase processing leading to the formation of the soluble, extracellular, secreted fragment, sAPPα. APP, and sAPPα, have important roles in neuroprotection, synaptic plasticity, neurite outgrowth, and synaptogenesis. Neuroprotection is a key physiological function of APP that involves regulation of calcium homeostasis and expression of survival genes and neurotrophic factors. It is now recognised that there is some commonality in the pathological cascades leading to neuronal death in ischaemia, traumatic brain injury, and neurodegeneration, particularly dysregulation of calcium homeostasis, and APP has been postulated to exercise its neuroprotective action by positively intervening in these pathways. By contrast, when the amyloidogenic pathway is dysfunctional, the secretase balance is disrupted, and β-secretase generates pathological amyloidβ peptides, which are important in the development of Alzheimer’s disease (Kogel et al. 2012; Plummer et al. 2016; Hefter and Draguhn 2017).

Signalment for the 6 PEM cases studied herein was: 4 year-old, Merino-Border Leicester cross ewe (20–01197); 3-year-old, Merino wether (20–01619); 5-year-old, Composite ewe (20–03187); 5-month-old, Merino wether (20–03565); 4-month-old ewe (breed not stated) (20–04893); and 4-year-old, Merino ewe (20–00037). The four control brains were collected from 18-month to 2-year-old, Merino-Border Leicester cross, clinically normal wethers.

The diagnosis of PEM was made on the basis of relevant clinical history and characteristic morphological changes of pseudolaminar cerebrocortical necrosis, with exclusion of water deprivation/intoxication, sources of lead and excess sulphur, and thiaminase-containing poisonous plants, all of which can produce a similar clinical presentation and pathological changes (Summers et al. 1995; Vandevelde et al. 2012; Cantile and Youssef 2016). Affected sheep were sometimes blind and showed aimless wandering, head pressing, nystagmus, bruxism, ptyalism, and muscle tremors. This progressed to recumbency and tonic-clonic convulsions. Brains from control and PEM cases were immersion-fixed in 10% neutral buffered formalin, coronal sections paraffin-embedded, and 6 μm sections cut and stained with H&E. The same area of temporal cortex was examined in PEM cases and control brains. A semi-quantitative analysis of neuronal APP immunopositivity in comparable neuroanatomical areas of laminar cortical necrosis, in each of the 6 PEM cases, was conducted by counting 100 neurons for the presence of APP-immunoreactivity occupying >75% of the perikaryon.

For immunohistochemistry, a mouse monoclonal antibody (gift from Colin Masters, clone 22C11) against APP was used. The epitope of the APP-specific monoclonal antibody 22C11 is located in the NH_2_-terminal, cysteine containing region of APP between residues 66 and 81 (Hilbich et al. 1993). In brief, sections were dewaxed using xylene and then rehydrated through alcohols. Sections were then treated with methanol/hydrogen peroxide for 30 min. All sections were then rinsed twice in phosphate buffered saline (PBS) (pH 7.4) for a further 5 min each wash. Antigen retrieval was performed using citrate buffer (pH 6) in a temperature calibrated microwave oven. Slides were then allowed to cool and washed twice in PBS. Non-specific proteins were blocked using normal horse serum for 30 min. Antibody was then applied at a dilution of 1:1000 at room temperature overnight. The following day, the sections were given two washes in PBS, then a biotinylated horse anti-mouse secondary (Vector Laboratories, cat. BA-2000) was applied for 30 min at room temperature. Following two PBS washes, the sides were incubated for 1 h at room temperature with a streptavidin-conjugated peroxidase tertiary (ThermoScientific, cat. 21,127). 5 μ sections were then visualised using 3–3′ diaminobenzidinetetrahydrochloride (DAB), washed, counterstained with haematoxylin, dehydrated, cleared and mounted on glass slides. For each batch of slides, a known positive control for the species of interest was run, with and without a minus primary control. An isotype control was also run. The internal positive control was APP-immunopositive axons, both amongst the red neurons and in the contiguous white matter of a PEM case, APP being the most sensitive, early marker of axonal injury (Blumbergs et al. 2008).

In order to precisely match individual H&E-stained red neurons in an area of laminar cerebrocortical necrosis to their corresponding APP-immunopositive neurons, the PEM region in all cases was first stained with H&E and then scanned. The cover slip was then removed and the slide rehydrated, placed in acid alcohol to remove the eosin counterstain, then immunostained for APP and scanned.

In 4 control brains collected from clinically normal sheep, cortical neurons were well-preserved (Fig. 1). All cortical neurons were APP-immunonegative (Fig. 1), except for an occasional neuron showing weak APP-immunopositivity, consistent with previous APP immunohistochemical studies in sheep (Van den Heuvel et al. 1999; Finnie et al. 2010). In PEM cases, red neurons showed varying degrees of cytoplasmic shrinkage, the more shrunken the cytoplasm, the more hypereosinophilic the H&E staining, and the more likely the nucleus was pyknotic; some neurons were triangular in shape or more elongated (Fig. 2). In all 6 PEM cases, a large majority of red neurons showed strong APP cytoplasmic immunolabelling, which occupied much of the cytoplasmic volume (  Fig.3). The percentage of neurons showing APP immunopositivity according to our mode of assessment in the 6 PEM cases was 95%, 93%, 92%, 91%, 88% and 86%. As shown in a representative section from case 20–03585 in Fig. 4, the same red neurons stained with H&E were also APP-immunopositive.

**Fig. 1:**
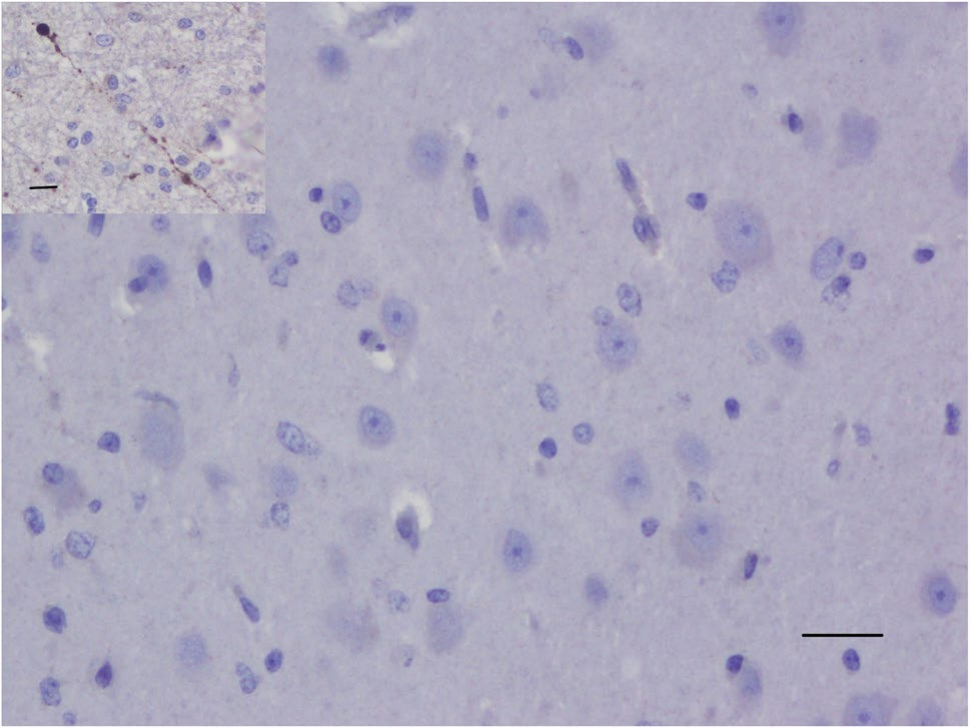
Clinically normal control sheep. Cerebrocortical neurons are well-preserved and APP-immunonegative. APP immunohistochemistry. Scale bar = 120 μm. Inset - APP internal positive control. APP-immunopositive injured axons in the white matter contiguous with pseudolaminar cerebrocortical necrosis in a PEM case, showing many varicosities (spheroids). Scale bar = 80 μm

**Fig. 2:**
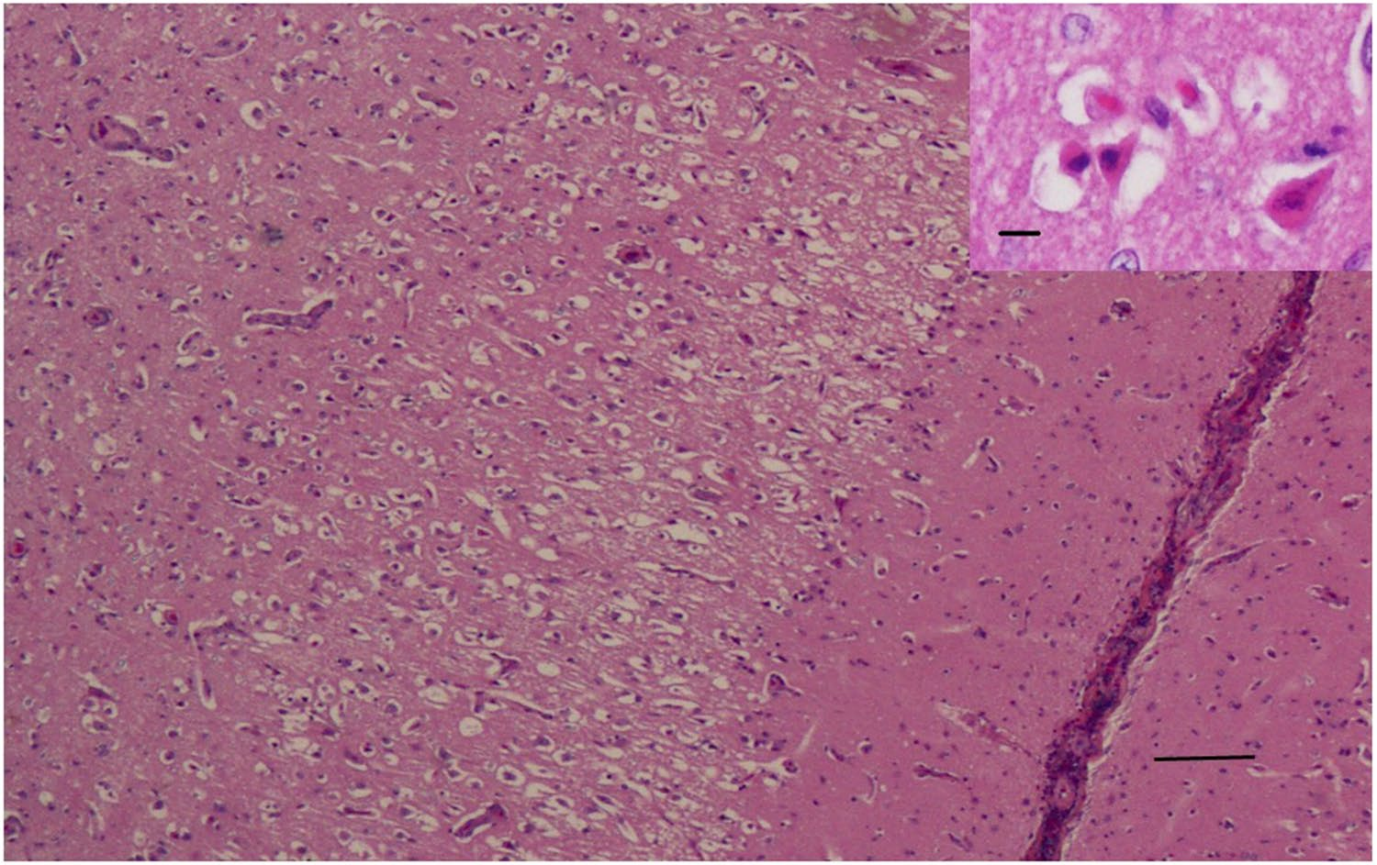
PEM case. Numerous red neurons in an area of pseudolaminar cerebrocortical necrosis. H&E. Scale bar = 60 μm. Inset - the neuronal cytoplasm is shrunken and hypereosinophilic, with large, clear perineuronal spaces. H&E. Scale bar = 160 μm

**Fig. 3:**
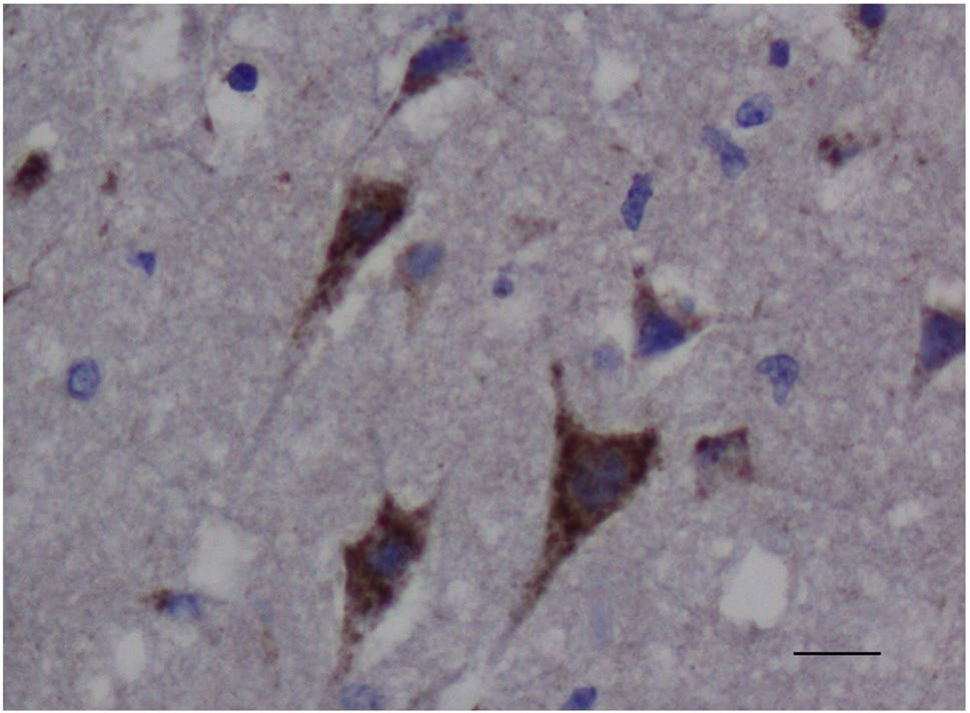
PEM case (20–03565). Red neurons show strong APP immunolabelling. Scale bar = 180 μm

**Fig. 4:**
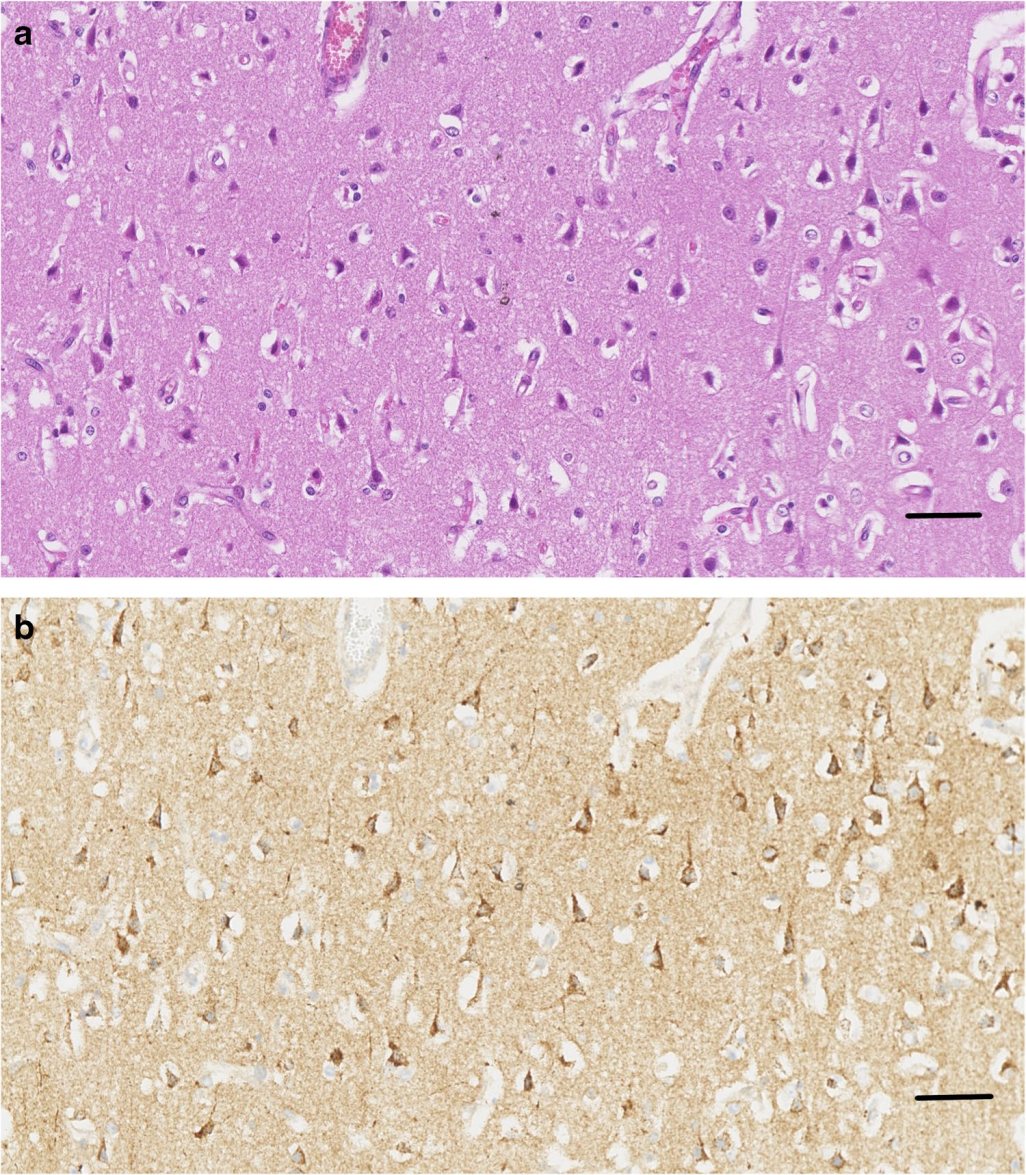
Representative image from an area of pseudolaminar cerebrocortical necrosis in case # 20–03585 showing that red neurons stained with H&E (A) were also APP-immunopositive (B) in the same section. Scale bar = 80 μm

Neuronal red cell change is generally considered to be an irreversible lesion (Vandevelde et al. 2012). However, the results of the present study suggest that some red neurons may not be irredeemably destined to die, but rather this change may be potentially reversible, with recovery of function. When neurons are stressed by injurious insults such as hypoxia, APP expression is upregulated as a neuroprotective response (Hefter and Draguhn 2017). Thus, while a proportion of red neurons in PEM cases will undoubtedly die, the widespread and robust increase in APP expression found in the vast majority of these neurons suggests that this neuroprotective reaction may lead to survival of at least some of these damaged cells.

Since neuronal red cell change and APP upregulation are dynamic, evolving processes, it would be very useful to follow the temporal course of their differential expression in an experimental animal model, but this would be difficult to achieve, particularly with ovine PEM. Temporal studies have, for example, demonstrated that dark neurons represent a reversible cellular alteration, with reversal of cytoplasmic condensation and preservation of organelles and cell membranes (Kalaria et al. 2015). The findings of the present study were confined to a single time-point, which was unknown in each of the diagnostic PEM cases examined. Furthermore, the time course of increased APP expression relative to the progressive development of neuronal red cell change could not be determined.

In conclusion, while neuronal APP upregulation can be neuroprotective, it remains speculative as to whether increased APP expression can prevent cell death and facilitate neuronal survival in PEM. It does, however, suggest that these neurons can potentially be rescued in both structure and function, rather than red cell change being regarded as an inevitable cell death event.

## Declarations

This used archival formalin-fixed paraffin-embedded brain tissue, collected during routine post mortem examination of sheep from commercial flocks. Brains were submitted to Agriculture Victoria Research, AgriBio Centre, for disease diagnosis and control. Animal ethics committee approval not required.
